# Constitutive Endocytosis and Turnover of the Neuronal Glycine Transporter GlyT2 Is Dependent on Ubiquitination of a C-Terminal Lysine Cluster

**DOI:** 10.1371/journal.pone.0058863

**Published:** 2013-03-06

**Authors:** Jaime de Juan-Sanz, Enrique Núñez, Beatriz López-Corcuera, Carmen Aragón

**Affiliations:** 1 Centro de Biología Molecular ‘‘Severo Ochoa’’, Universidad Autónoma de Madrid, Consejo Superior de Investigaciones Científicas, Madrid, Spain; 2 Centro de Investigación Biomédica en Red de Enfermedades Raras (CIBERER), ISCIII, Madrid, Spain; 3 IdiPAZ-Hospital Universitario La Paz, Madrid, Spain; University of São Paulo, Brazil

## Abstract

Inhibitory glycinergic neurotransmission is terminated by sodium and chloride-dependent plasma membrane glycine transporters (GlyTs). The mainly glial glycine transporter GlyT1 is primarily responsible for the completion of inhibitory neurotransmission and the neuronal glycine transporter GlyT2 mediates the reuptake of the neurotransmitter that is used to refill synaptic vesicles in the terminal, a fundamental role in the physiology and pathology of glycinergic neurotransmission. Indeed, inhibitory glycinergic neurotransmission is modulated by the exocytosis and endocytosis of GlyT2. We previously reported that constitutive and Protein Kinase C (PKC)-regulated endocytosis of GlyT2 is mediated by clathrin and that PKC accelerates GlyT2 endocytosis by increasing its ubiquitination. However, the role of ubiquitination in the constitutive endocytosis and turnover of this protein remains unexplored. Here, we show that ubiquitination of a C-terminus four lysine cluster of GlyT2 is required for constitutive endocytosis, sorting into the slow recycling pathway and turnover of the transporter. Ubiquitination negatively modulates the turnover of GlyT2, such that increased ubiquitination driven by PKC activation accelerates transporter degradation rate shortening its half-life while decreased ubiquitination increases transporter stability. Finally, ubiquitination of GlyT2 in neurons is highly responsive to the free pool of ubiquitin, suggesting that the deubiquitinating enzyme (DUB) ubiquitin C-terminal hydrolase-L1 (UCHL1), as the major regulator of neuronal ubiquitin homeostasis, indirectly modulates the turnover of GlyT2. Our results contribute to the elucidation of the mechanisms underlying the dynamic trafficking of this important neuronal protein which has pathological relevance since mutations in the GlyT2 gene (*SLC6A5*) are the second most common cause of human hyperekplexia.

## Introduction

Inhibitory glycine neurotransmission is terminated by specific transporters, GlyTs (GlyT1 and GlyT2), which mediate the reuptake of glycine from the synaptic cleft. GlyTs belong to the neurotransmitter:sodium symporter family (SLC6 gene family), which includes transporters for most of the neurotransmitters (serotonin, dopamine, norepinephrine and GABA) in the central nervous system (CNS) [1]. By mediating the synaptic recycling of glycine, the neuronal transporter GlyT2 preserves the quantal glycine content in synaptic vesicles and assists GlyT1 in regulating glycine levels at the synaptic cleft. Gene deletion studies suggest that modification of glycine transporter activity may be beneficial in several human disorders, including neuromotor deficiencies (startle disease, myoclonus), pain and epilepsy [2]–[4]. Indeed, missense, nonsense, frameshift, and splice site mutations in the gene encoding GlyT2 can induce hyperekplexia in humans and congenital muscular dystonia type 2 (CMD2) in Belgian Blue cattle [5]–[9]. In addition, a microdeletion in SLC6A5 as cause of startle disease in Irish Wolfhounds has been reported [10].

Protein trafficking plays a fundamental role in the control of neuronal activity and it has been identified as a primary regulatory mechanism for several plasma membrane neurotransmitter transporters, providing a rapid means to modulate their activity [11]. The surface expression of GlyT2 is controlled by a variety of stimuli that influence its trafficking, including PKC and syntaxin 1A [12], [13]. GlyT2 is recycled between the cell surface and the cell interior along constitutive and PKC-regulated trafficking pathways [14], and a large proportion of GlyT2 resides in intracellular endosomal membranes of rat brainstem neurons and heterologous cells under steady-state conditions [15], [16].

Recent findings have demonstrated the importance of ubiquitination in the endocytosis of several membrane proteins, suggesting that the attached ubiquitin molecule may act as a platform for the recruitment of the clathrin-dependent endocytic machinery [17], [18]. In fact, ubiquitination is the mechanism proposed to mediate PKC-dependent endocytosis of neurotransmitter transporters [19]–[22]. Accordingly, we recently demonstrated that clathrin-mediated endocytosis is the main mechanism driving constitutive and regulated GlyT2 internalization and the lysine 791 in the C-terminal tail of GlyT2 was proposed to be the major determinant of PKC-induced internalization [23]. However, the role of ubiquitination in the constitutive endocytosis of membrane neurotransporters is less clear. Indeed, the dopamine transporter DAT is constitutively internalized in an ubiquitination-independent manner [24], while the constitutive endocytosis of GlyT1 and the glutamate transporter GLT1 requires the ubiquitination of several lysines. Hence, it appears that the requirement of ubiquitination is not a general condition for constitutive endocytosis of these transporters [19], [25].

In the present study, we investigated the possible role of ubiquitination in the constitutive internalization of neuronal GlyT2 and its sorting to recycling and/or degradation pathways. Our results show that constitutive endocytosis of GlyT2 is dependent on the ubiquitination of the cytoplasmic C-terminal lysine cluster (K751, K773, K787 and K791). The dynamic ubiquitination/deubiquitination process controls GlyT2 turnover through constitutive sorting mainly to the recycling pathway and targeting the transporter primarily to the degradation pathway via PKC-mediated ubiquitination. In neurons, the ubiquitination status of GlyT2 is highly responsive to the free ubiquitin pool, which is mainly controlled by UCHL1 deubiquitinase [26], [27]. Thus, UCHL1 activity may indirectly modulate the turnover of neuronal GlyT2. These findings demonstrate the requirement of ubiquitination in the regulation of neuronal GlyT2, a key protein in the physiology and pathology of glycinergic neurotransmission.

## Materials and Methods

### Materials

Male wistar rats were bred under standard conditions at the Centro de Biología Molecular Severo Ochoa. All animal work performed in this study was carried out in accordance with procedures approved in the Directive 86/609/EEC of the European Union with approval of the Ethics Committee of the Universidad Autónoma de Madrid. PYR-41 (4-[4-(5-nitro-furan-2-ylmethylene)-3,5-dioxo-pyrazolidin-1-yl]-benzoic acid ethyl ester), and inhibitors of UCH-L1 [LDN-57444 (LDN)] and ubiquitin C-terminal hydrolase-L3 (UCH-L3) (4, 5, 6, 7-tetrachloroindan-1,3-dione, TCID) were purchased from Calbiochem (San Diego, CA). All other chemicals were purchased from Sigma-Aldrich. Antibodies against GlyT2 (rabbit and rat: [28], [16]), calnexin (Stressgen), syntenin-1 (Abcam), tubulin (Sigma-Aldrich) and syntaxin1A and Ubiquitin (Clone P4D1) (Santa Cruz) were used. Agarose-conjugated anti-multiubiquitin (monoclonal antibody, clone FK2) was purchased from MBL International. Fluorophore-coupled secondary antibodies were acquired from Molecular Probes. Multiple sequence alignment was performed with CLUSTAL 2.1 multiple sequence alignment software, using the rat GlyT2 sequence as the query at www.ebi.ac.uk.

### Primary cultures of neurons

The brainstem and spinal cord from 16-day-old Wistar rat fetuses or the hippocampus from 18-day-old Wistar rat fetuses were isolated in Hank's Balanced Salt Solution buffer (Invitrogen), dissociated with trypsin as described previously [29] and grown in culture plates. After 2 days, cytosine arabinoside (2.5 µM) was added to inhibit further glial growth and the primary neurons were studied after 14 days in culture.

### Transfection of MDCK cells and hippocampal neurons

Madin – Darby canine kidney II (MDCK II) cells (American Type Culture Collection) were grown at 37°C and 5% CO_2_ in minimal essential medium supplemented with 10% fetal bovine serum. Transient expression was achieved using Lipofectamine™ 2000 (Invitrogen) following the manufacturer's instructions. Reproducible results were obtained with 50–60% confluent cells in a 60 mm dish using 6 μg of total DNA. The cells were incubated for 48 h at 37°C before the experiment was performed. Hippocampal neurons were obtained as described above. The cells (DIV10) were transfected with Effectene (Qiagen) following manufacturer's instructions using 10 μl of Effectene per 1 μg of DNA in a 12 mm dish.

### Generation of mutants

Substitution mutants were generated with the QuikChange Site-Directed Mutagenesis kit (Stratagene), using the rat GlyT2 in pCDNA3 as described previously [29]. The 4KR mutant (GlyT2 mutant in which all the C-terminal lysines are substituted by arginines) was created via four consecutive rounds of Polymerase Chain Reaction (PCR) site-directed mutagenesis, one for each C-terminal lysine-to-arginine mutation. All point mutations were verified by sequencing.

### Immunocytochemistry and confocal imaging

MDCK II cells and hippocampal neurons were grown on glass coverslips and transfected with the corresponding expression vectors as indicated above. Immunostaining was performed as described previously [23] and the cells were visualized by confocal microscopy on a Microradiance microscope (BioRad) using a vertical Axioskop 2 microscope (Zeiss), or with a LSM510 META confocal microscope coupled to an inverted AXIOVERT 200 microscope (Zeiss). IMAGEJ (National Institutes of Health) software and LSM image browsers (Carl Zeiss Inc.) were used for image processing.

### Anti-multiubiquitin immunoprecipitation

Brainstem and spinal cord primary neurons (100 µg) or MDCK II cells were lysed for 30 min at room temperature (RT) at a concentration of 1 mg of protein/ml in TN buffer (25 mM TrisHCl and 150 mM NaCl, pH 7.4) containing 0.25% Nonidet P-40 (NP-40), 50 mM N-ethylmaleimide and protease inhibitors (PIs: 0.4 mM phenylmethylsulfonyl fluoride [PMSF] + Sigma cocktail). Agarose-conjugated anti-multiubiquitin (12 μl) was added and incubated for 1 h at RT. The beads were collected by mild centrifugation and washed 3 times for 5 minutes with lysis buffer. Finally, the beads were pelleted and the ubiquitinated proteins were eluted in Laemmli buffer at 75°C for 10 min, resolved in sodium dodecyl Sulfate Polyacrylamide Gel electrophoresis (SDS/PAGE) gels (7.5%), detected in Western blots with enhanced chemiluminescence (ECL) and quantified on a GS-710 calibrated imaging densitometer (Bio-Rad).

### Cell surface labelling with Sulfo-NHS-SS-biotin

Surface proteins of transfected MDCK II cells or primary brainstem and spinal cord neurons (14 DIV) were washed with 1.0 ml of Phosphate Buffered Saline (PBS) at 4°C and incubated for 40 min at 4°C with 1 mg/ml of non-permeable sulfo-NHS-SS-biotin reagent (Thermo Fisher Scientific) in PBS. Cells were then washed 3 times with 1 ml of the same solution supplemented with 100 mM lysine and scraped in 50 mM Tris-HCl [pH 7.4], 150 mM NaCl (TN) buffer plus 0.4 mM phenylmethylsulfonylfluoride (PMSF) and protease inhibitor mixture (Sigma). Total proteins were solubilized for 30 min at 4°C in RIPA buffer (150 mM NaCl, 5 mM EDTA, 1% Triton X-100, 1% SDS and 0.25% sodium deoxycholate). Streptavidin-agarose beads (40 μl per sample: Sigma) were added and incubated for 1 h at 4°C with agitation. Bead-bound biotinylated proteins (B) were eluted for 15 min at 70°C with Laemmli buffer (40 mM Tris [pH 6.8], 2% SDS, 10% glycerol, 0.1 mM dithiothreitol and 0.01% bromophenol blue). Total protein (T; 10 μg) and biotinylated protein (B; 30 μg) were run on a 7.5% SDS-polyacrylamide gel and after analyzed by Western blot with specific GlyT2 antibodies, the bands were visualized by ECL and quantified in the linear range on a GS-710 calibrated imaging densitometer (Bio-Rad) with Quantity One software. Calnexin immunoreactivity was used as a non-biotinylated protein control. The standard error of the mean (S.E.M) was calculated after densitometric analysis of at least three separate experiments.

### Glycine transport assay

Transport assays in MDCK cells were performed at 37°C in PBS plus 10 mM glucose, containing 2 μCi/ml [^3^H]-labelled glycine (1.6 TBq/mmol; PerkinElmer) diluted to a final glycine concentration of 10 μM, as described previously [23]. Reactions were terminated after 10 min by aspiration and transport was quantified by subtracting the glycine accumulated in mock-transfected MDCK cells from that of the transporter-transfected cells and normalized to the protein concentration.

### Quantification of co-localization and cell surface rates from immunofluorescence microscopy images

To perform the quantification of co-localization, Pearsońs value was analyzed with IMAGEJ software (National Institutes of Health), using at least 30 images for each condition. Images were processed with a 2.0 pixel median filter, and threshold used was automatically determined by JACoP plugin [30]. Pearsońs value was obtained with JACoP by comparing the two thresholded channels and measuring the correlation between them. The value can range from -1 to 1, being 1 the maximal co-localization possible (two identical images), and usually values from 0.5 to 1.0 can be considered as a valid co-localization [31]. The quantification of cell surface GlyT2 was performed as described previously [23].

### Data Analysis

All statistical analyses were performed using SPSS 19.0 (SPSS Inc., Chicago, IL) and graphs and curves were generated with Origin 8.0 (OriginLab Corp, MA). One-way analysis of variance (ANOVA) was used to compare multiple groups, with subsequent Tukey's post-hoc test to determine the significant differences between samples. The Student's *t*-test was used to compare two separate groups. p values <0.05 were considered significant.

## Results

### The constitutive endocytosis of GlyT2 is regulated by ubiquitination of its C-terminal lysines

Ubiquitination is a well-described post-translational modification involved in the PKC-induced endocytosis of various membrane proteins, including dopamine, glutamate and glycine transporters (DAT, GLT1, GlyT1b, GlyT2). However, its role in the constitutive endocytosis of these proteins is less understood. We recently demonstrated a direct relationship between the ubiquitination of lysine 791 in the C-terminus of the neuronal glycine transporter GlyT2 and its PKC-dependent endocytosis. Hence, in the present study we investigated whether the constitutive endocytosis of GlyT2 was also ubiquitin-dependent, initially using the MDCK cell line, a suitable background for our immunofluorescence studies due to the efficient and uniform expression of transfected GlyT2 at the cell surface of these cells, as described previously [23]. To study the constitutive endocytosis of GlyT2 we used the cationophore monensin, an experimental strategy commonly employed to analyze the constitutive endocytosis of membrane proteins. Monensin is an inhibitor of transport via acidic endosomal compartments and thus, interferes with the intracellular trafficking of proteins. Monensin does not prevent endocytosis but rather, by blocking protein recycling to the plasma membrane and likely degradation, it promotes the accumulation of endocytosed membrane proteins [23], [32]–[34].

**Figure 1:**
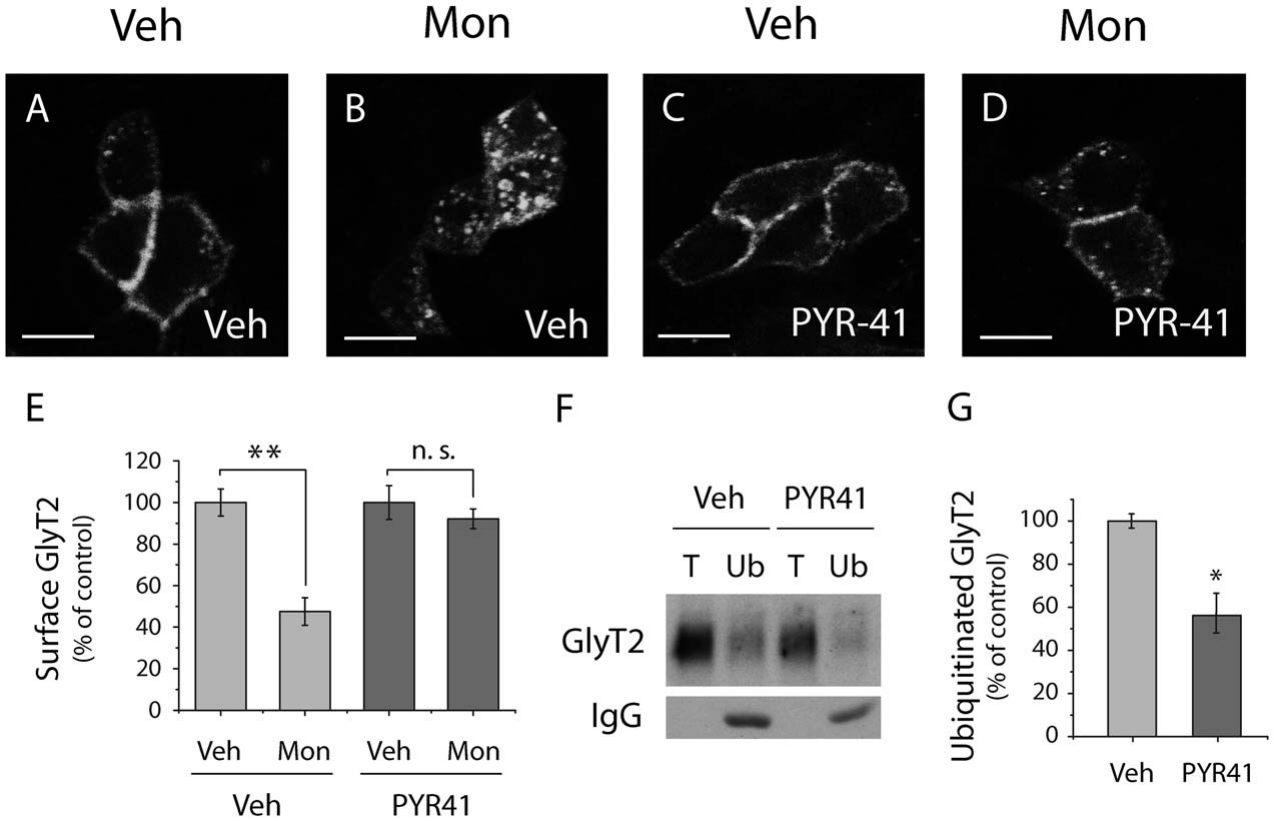
Pharmacological inhibition of the E1 ubiquitin-activating enzyme activity reduces constitutive GlyT2 endocytosis. A–D) MDCK cells were transfected to express wild-type GlyT2, and were preincubated for 2 h with vehicle (A, B) or PYR-41 (50 μM) (C, D). Then were incubated for 30 min with vehicle (A, C) or monensin (35 μM) (B, D) maintaining the previous pretreatment. The cells were then fixed with 4% paraformaldehyde, immunostained to visualize GlyT2 and analyzed by confocal microscopy. Scale bar  = 15 µm. Note the reduction in constitutively endocytosed GlyT2 in the presence of monensin and PYR-41 (D). E) Quantification of GlyT2 fluorescence intensity at the cell surface was performed as described in Materials and Methods. The histogram represents the mean ± SEM (n = 4; on average, 30 cells per condition were analyzed in each experiment). **, significantly different, p<0.01 by ANOVA with Tukey's post hoc test. n.s., not statistically significant. F) MDCK cells expressing wild-type GlyT2 were incubated with PYR-41 or the vehicle alone, as described above, the cells were lysed and the ubiquitinated transporters were immunoprecipitated with agarose-conjugated anti-multiubiquitin antibody. The immunoprecipitates were probed with an anti-GlyT2 antibody. Ub, anti-multiubiquitin immunoprecipitation (75 μg); T, total protein (10 μg). G) Quantification of three experiments performed as described in (F). Bars represent the mean ± SEM levels of PYR-41-treated ubiquitinated transporter relative to those of ubiquitinated transporter in control cells exposed to the vehicle alone. *, significant difference relative to controls; p<0.05 (Student's t-test).

We first examined the effect of PYR41, a cell-permeable specific inhibitor of the E1 ubiquitin-activating enzyme that catalyzes an initial and critical step in the protein ubiquitination pathway [35]. As expected, monensin promoted the intracellular accumulation of GlyT2 (Fig. 1A, B) with a concomitant marked decrease in protein levels in the plasma membrane (Fig. 1B, E). When cells were pretreated with PYR-41 (50 μM) and then incubated with monensin (35 μM), a considerable reduction of GlyT2 endocytosis with a substantial amount of the transporter remaining at the cell surface was observed (Fig. 1D, E). Immunoprecipitation experiments provided direct evidence that inhibition of GlyT2 ubiquitination underlies the reduction in GlyT2 endocytosis (Fig. 1F, G). MDCK cells expressing wild-type GlyT2 were maintained in the presence or absence of PYR41 and the ubiquitinated transporter was immunoprecipitated from cell lysates with agarose-conjugated anti-multiubiquitin (clone FK2), an antibody that recognizes poly- and mono-ubiquitinated proteins [36], and samples were then analyzed by Western blot with anti-GlyT2 antibodies. Indeed, the inhibition of E1 ubiquitin-activating enzyme by PYR41 clearly reduced the amount of ubiquitinated GlyT2 (56.97±6.30% SEM, Fig. 1G), indicating a positive correlation between ubiquitination and constitutive GlyT2 internalization.

**Figure 2:**
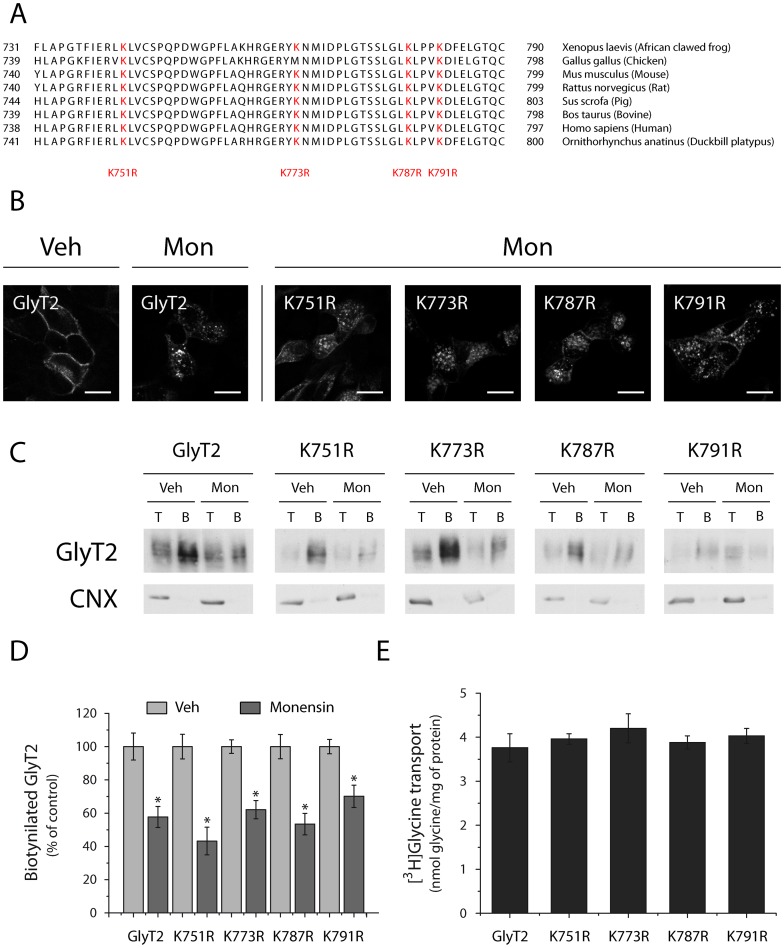
Mutation of each lysine of the GlyT2 C-terminal does not impair GlyT2 constitutive endocytosis. A) Multiple sequence alignment of rat GlyT2 C-terminus region (740–799) from different species was obtained with the CLUSTAL 2.1 multiple sequence alignment method. Identical conserved lysines from different species are shown in red. B-C) MDCK cells expressing wild-type GlyT2 or one of four different point mutants (K751R, K773R, K787R or K791R) were exposed for 30 min to monensin (35 μM) at 37°C or the vehicle alone, fixed with 4% paraformaldehyde, immunostained to visualize GlyT2 and analyzed by confocal microscopy. To simplify the figure, only the wild-type GlyT2 control (Veh) is displayed (all other controls were comparable). Scale bar  = 15 μm. C) Representative immunoblot of MDCK cells expressing wild-type GlyT2 or the indicated mutants. Cells were treated with monensin or the vehicle alone, as described above. The cell surface proteins were labeled with sulfo-NHS-SS-biotin and biotinylated proteins were pulled down with streptavidin-agarose beads. GlyT2 expression was analyzed in Western blots and calnexin immunodetection was used as a non-biotinylated protein control. B, biotinylated protein (30 μg); T, total protein (10 μg). D) Densitometric analysis of three independent Western blots as shown in (C), relative to the control values (Veh). E) [^3^H]-Glycine uptake during 10 minutes was measured in MDCK cells expressing wild-type GlyT2 or the mutants indicated and transport activity is denoted in nmol of glycine/mg of protein. The data represent the means ± SEM and no significant differences respect to vehicle were observed performing ANOVA analysis (with Tukey's post-hoc test).

**Figure 3:**
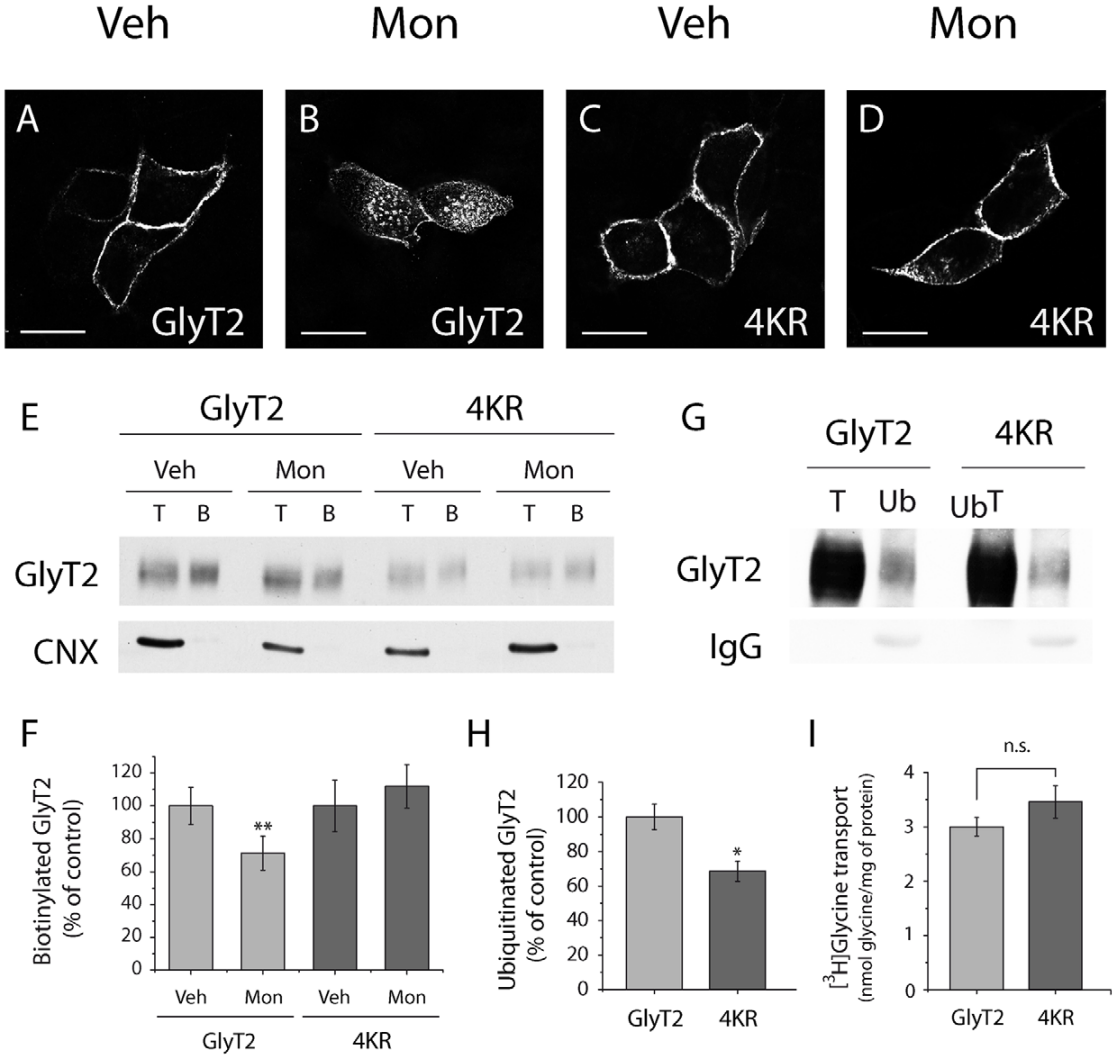
The 4KR GlyT2 mutant exhibits impaired endocytosis and lower basal ubiquitination than wild-type GlyT2. A–D) MDCK cells were transfected with wild-type GlyT2 or with 4KR mutant cDNAs (GlyT2 with lysines in positions 751, 773, 787 and 791 mutated to arginines). After 48 h the cells were exposed for 30 min to monensin (35 μM) at 37°C or the vehicle alone, fixed with 4% paraformaldehyde, immunostained to visualize GlyT2 and analyzed by confocal microscopy. Scale bar  = 15 μm. Note that endocytosis of the 4KR mutant is blocked in the presence of monensin (D). E) Representative immunoblot of MDCK cells expressing wild-type GlyT2 or the 4KR mutant. Cells were treated for 30 min with the vehicle alone or with monensin (35 μM) at 37°C. Cell surface proteins were labeled with sulfo-NHS-SS-biotin and the biotinylated proteins were pulled down with streptavidin-agarose beads. GlyT2 expression was analyzed in Western blots and calnexin immunodetection was used as a non-biotinylated protein control. B, biotinylated protein (30 μg); T, total protein (10 μg). F) Densitometric analysis of four independent Western blots as in (E) relative to the control values (veh). Data represent means ± SEM. **, significant difference with respect to control, p<0.01 (ANOVA with Tukey's post-hoc test). Note that constitutive endocytosis of the 4KR mutant is blocked in the presence of monensin. G) MDCK cells expressing wild-type GlyT2 or the 4KR mutant were lysed, ubiquitinated proteins were immunoprecipitated with agarose-conjugated anti-multiubiquitin antibodies, and GlyT2 was analyzed in Western blots. Ub, anti-multiubiquitin immunoprecipitation (50 μg); T, total protein (10 μg). H) Quantification of four experiments performed as described in (G). Bars represent the mean ± SEM of the amount of 4KR mutant ubiquitinated transporter relative to the amount of wild-type ubiquitinated transporter. *, significant difference with respect to control, p<0.05 (Student's t-test). I) [^3^H]-Glycine uptake during 10 minutes was measured in MDCK cells expressing wild-type GlyT2 or the 4KR mutant and transport activity is denoted in nmol of glycine/mg of protein. The data represent the means ± SEM and no significant differences respect to vehicle were observed performing student's t-test.

Lysine clusters in different cytosolic domains have been identified as ubiquitination sites required for endocytosis of GLT1 [20] and DAT [17] neurotransporters. To investigate this phenomenon in GlyT2, we first examined the contribution of each residue (K751, K773, K787 and K791) present in the C-terminus of GlyT2 to its constitutive endocytosis by immunofluorescence confocal microscopy and biotinylation assays. These GlyT2 lysine residues are evolutionarily conserved in most animal species (as evident in sequence alignments: Fig. 2A, in red), suggesting an important role in GlyT2 function. Mutants in which each of the four lysine residues was substituted individually to arginine displayed similar constitutive internalization to that observed for the wild-type protein when assessed by confocal microscopy (Fig. 2B). Likewise, quantitative biotinylation revealed similar levels of remaining transporter at the cell surface in the presence of monensin for each mutant and the wild-type transporter (Fig. 2C, D), suggesting that ubiquitination of the individual K751, K773, K787 and K791 residues contributed weakly to GlyT2 constitutive endocytosis. [^3^H]-glycine uptake assays depicted in fig. 2E showed similar transport activity levels for every mutant and wild-type GlyT2. Next, we generated a GlyT2 mutant in which all four C-terminal lysines are simultaneously substituted by arginines (4KR mutant) and the constitutive endocytosis was assayed as above. As immunofluorescence pictures show, in contrast to wild-type GlyT2, the 4KR mutant predominantly remained at the cell surface in the presence of monensin, suggesting a role for the C-terminal lysine cluster in the constitutive internalization of the transporter (Fig. 3A–D). These findings were further supported by quantification of transporter levels at the cell surface by biotinylation (Fig. 3E, F). In addition, immunoprecipitation experiments provided direct evidence that lysine cluster ubiquitination is a critical requirement for the constitutive endocytosis of GlyT2 (Fig. 3G, H). Fig. 3G and H shows that 4KR mutant was around 32% less ubiquitinated than the wild-type transporter but it still remained ubiquitinated. Redundancy in ubiquitination sites has been frequently reported [33], such that other lysines are ubiquitinated in the absence of the main conjugation sites. This may account for the residual ubiquitination of the 4KR mutant. Alternatively, other intracellular lysine residues may be ubiquitinated, but in either case its contribution to the constitutive endocytosis does not appear to be functionally relevant. [^3^H]-glycine uptake assays showed similar transport activity for 4KR mutant and wild-type GlyT2 (Fig. 3I). Together, these results point to C-terminal K751, K773, K787 and K791 of GlyT2 as targets of the ubiquitination required for its constitutive endocytosis.

### Neuronal localization of GlyT2 is not impaired by substitution of the C-terminal lysine cluster

**Figure 4:**
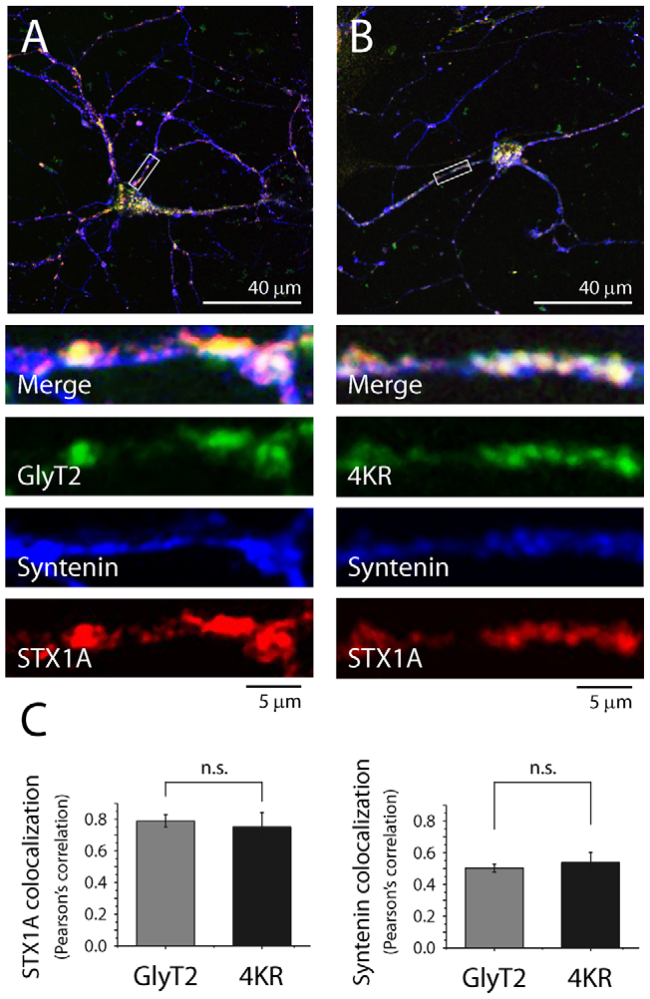
Mutation of the C-terminal lysine cluster of GlyT2 does not impair its neuronal localization in transfected hippocampal neurons. A–C) Hippocampal neurons were transfected with wild type GlyT2 or with the 4KR mutant at 10 DIV. Three days after transfection, the cultures were fixed in pre-cooled 100% methanol at −20°C and stained for GlyT2 (green), syntaxin1A (red) and syntenin-1 (blue) specific antibodies. General scale bar, 40 μm. Scale bar in detailed images, 5 μm. C) Quantification of co-localization between syntaxin1A and GlyT2, or between syntenin-1 and GlyT2, was performed using Pearson's value of correlation as described in Materials and Methods. Note there is no significant difference in the neuronal localization between GlyT2 and 4KR mutant, by Student's T-test. The histogram represents the mean ± SEM (n = 3; on average, 30 images per condition were analyzed in each experiment).

Regarding the synaptic localization of the transporter, C-terminus region of GlyT2 has been suggested to be important for synaptic localization of the transporter since modification of its C-terminal type III PDZ domain binding motif reduces co-localization of GlyT2 with different synaptic markers [37]. To test if the C-terminal lysine cluster was also implicated in the neuronal localization of transporter we transfected primary hippocampal neurons with GlyT2 wild-type and 4KR mutant constructs and measured co-localization with syntenin-1, a PDZ domain protein, and syntaxin1A, a Soluble NSF Attachment Protein REceptor (SNARE) complex component (Fig. 4) We have selected syntenin-1 and syntaxin1A because they are the only proteins whose interaction with GlyT2 has been described in nervous tissue preparations [38], [13]. In agreement with previous results [37] no GlyT2 immunostaining was detected in non-transfected neurons (data not shown), indicating that the fluorescence was derived from the exogenous constructs of GlyT2. As figure 4 shows, both GlyT2 and 4KR present similar punctate distribution pattern (Fig. 4A–B) and similar levels of co-localization with syntaxin1A and sintenin-1 (Fig. 4C), indicating that the C-terminal lysine cluster appears not to be implicated in the synaptic localization of GlyT2. Together these results suggest that simultaneous substitution of C-terminal K751, K773, K787 and K791 residues does not affect GlyT2 interactions with syntaxin1A and syntenin-1, and therefore neither the proper neuronal localization of GlyT2.

### Ubiquitination modulates the intracellular distribution and degradation of GlyT2

**Figure 5:**
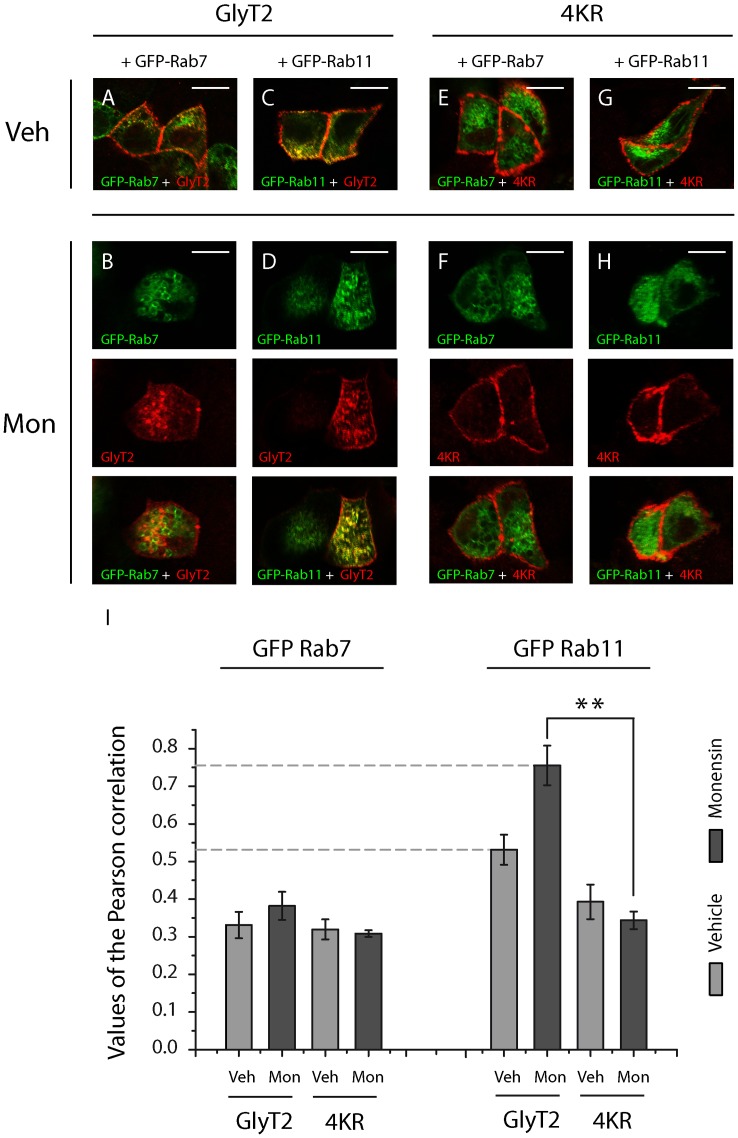
Endocytosed GlyT2 co-localizes with GFP-Rab11, whereas the 4KR mutant fails to internalize and accumulates in the surface. A-H) MDCK cells were transfected with wild-type GlyT2 and GFP-Rab7 (A, B) or GFP-Rab11 (C, D), or with the 4KR mutant and GFP-Rab7 (E, F) or GFP-Rab11 (G, H). After 48 h the cells were incubated for 30 min with monensin (35 μM) or the vehicle alone and fixed with 4% paraformaldehyde. GFP-Rab7 and GFP-Rab11 were visualized by GFP fluorescence (green), whereas GlyT2 was detected with the anti-GlyT2 antibody (shown in red). Note the increase in intracellular vesicles in monensin-treated cells expressing wild-type GlyT2 (B, D), which colocalizes with GFP-Rab11 (D) but not GFP-Rab7 (B). This increase was not observed in cells expressing the 4KR mutant transporter (F, H). Scale bar  = 15 μm. I) Quantification of colocalization using Pearsońs value was performed as described in Materials and Methods. Note the increase of colocalization between Rab11 and wild type GlyT2 in the presence of monensin, which cannot be observed with 4KR mutant. The histogram represents the mean ± SEM (n = 3; on average, 30 images per condition were analyzed in each experiment).**, significantly different, p<0.01 by ANOVA with Tukey's post hoc test.

To further investigate whether deficient ubiquitination alters the intracellular localization of GlyT2 we co-expressed wild-type or 4KR GlyT2 with different Rab proteins fused to green fluorescent protein in MDCK cells. Small Rab GTPases are well-known organizers of intracellular trafficking of membrane proteins in eukaryotic cells and they serve as markers of distinct endosomal compartments [39]. We previously showed that at steady state, most intracellular GlyT2 resides in a subset of Rab11-positive recycling endosomes in nerve terminals and neurons [16]. Accordingly, double immunofluorescence images revealed that at the steady state also in MDCK cells, intracellular GlyT2 mainly co-localized with Green Fluorescent Protein (EGFP)-Rab11, a marker of slow recycling endosomes. By contrast, we observed a small co-localization with EGFP-Rab7, a marker of late endosomes (Fig. 5A, C, I). The 4KR mutant accumulated on the cell surface and no intracellular vesicles expressing this transporter were detected (Fig. 5E, G). Moreover, the abundant EGFP-Rab11 and GlyT2-positive vesicles observed after 30 min of monensin treatment, suggest that GlyT2 is constitutively mainly sorted to the slow recycling pathway (Fig. 5D, I). In accordance with the reduced endocytosis of the 4KR mutant, only a few vesicles were observed containing both the 4KR transporter and EGFP-Rab11 (Fig. 5H, I).

**Figure 6:**
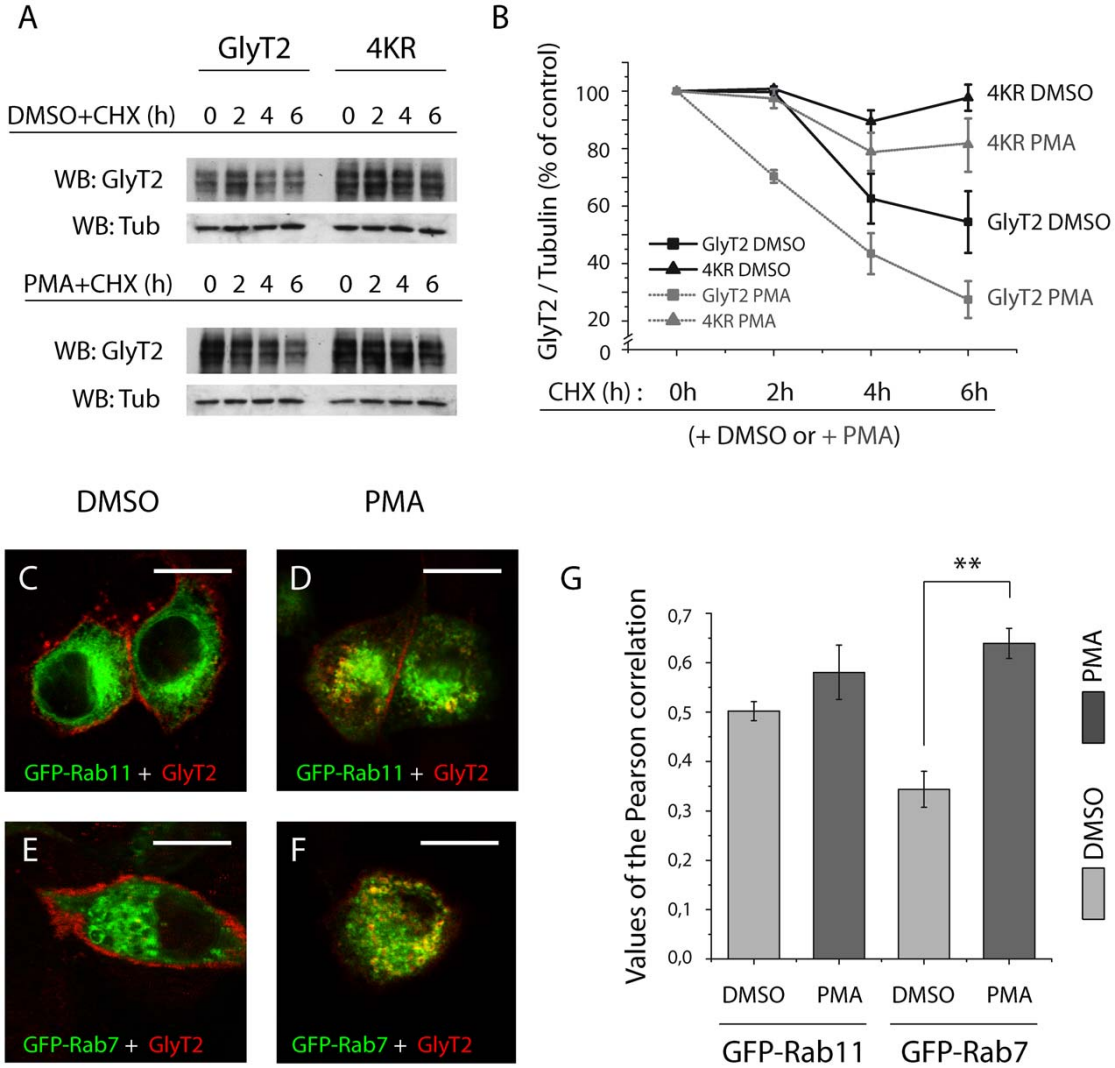
Reduction of GlyT2 C-terminal ubiquitination increases its constitutive stability and attenuates PMA-induced degradation. A) Representative immunoblot of MDCK cells expressing wild-type GlyT2 or the 4KR mutant. Cells were treated with cycloheximide (CHX: 10 μg/ml) or CHX plus PMA (1 μM) for the times indicated. The cells were then harvested, lysed and whole cell lysates (20 μg/lane) were resolved by SDS-PAGE and analyzed by immunoblotting with the anti-GlyT2 antibody. As a loading control, blots were reprobed with anti-tubulin. B) Degradation curves were generated by measuring GlyT2 band densities and normalizing them to corresponding tubulin band densities, designating time zero as 100%. Black curves correspond to CHX + DMSO conditions and grey curves correspond to CHX + PMA conditions. Densitometric analysis of three independent Western blots as in (A) was performed with the data representing the means ± SEM. C–F) MDCK cells were transfected with wild-type GlyT2 and GFP-Rab11 (C, D) or GFP-Rab7 (E, F). After 48 h the cells were incubated for 2 hours with PMA (1 μM) or the vehicle and fixed with 4% paraformaldehyde. GFP-Rab7 and GFP-Rab11 were visualized by GFP fluorescence (green), whereas GlyT2 was detected with the anti-GlyT2 antibody (shown in red). Note the increase in intracellular vesicles in PMA-treated cells expressing wild-type GlyT2 (D, F) which colocalizes with GFP-Rab7 (F). Scale bar  = 15 μm. G) Quantification of colocalization using Pearsońs value was performed as described in Materials and Methods. The histogram represents the mean ± SEM (n = 3; on average, 30 images per condition were analyzed in each experiment). **, significantly different, p<0.01 by ANOVA with Tukey's post hoc test.

In recent years, the importance of ubiquitination in controlling endocytosis and degradation of plasma membrane proteins has been established. Thus, we investigated whether ubiquitination affects the stability of GlyT2. We compared the degradation rate of wild-type GlyT2 and the ubiquitin-deficient 4KR mutant in untreated and PMA-treated cells in the presence of cycloheximide, a blocker of protein synthesis (Fig. 6). As we recently reported that PKC-dependent GlyT2 endocytosis is mediated by increased GlyT2 ubiquitination [23], PMA was used to establish the opposite scenario to that seen in the ubiquitin-deficient 4KR mutant. Quantification of Western blots revealed that the wild type protein decreased rapidly after 2 hours, with only 54.44±9.72% of initial protein remaining after 6 h. By contrast, the 4KR mutant protein was more stable and persisted longer in these cells. Exposure to Phorbol 12-Myristate 13-Acetate (PMA) markedly accelerated the degradation of GlyT2 and after 6 h, protein levels had fallen by 73.56±6.39% of initial amount (Fig. 6A, B). Increased degradation by PMA has been previously observed for DAT which is completely degraded within 2 h of PKC activation [40]. In accordance with the absence of the major target of PKC-induced ubiquitination (lysine 791, [23]), the 4KR mutant was stable through 2 h of PMA treatment. After longer PMA exposure time a small degradation was observed (Fig. 6A, B), probably as result of ubiquitination of other intracellular lysine residues when K791 is absent, as indicated above (see comments to Fig. 3G, H). Indeed, immunofluorescence assays showed a significant increase in GlyT2 and EGFP-Rab7 colocalization after PMA treatment (Fig. 6F, G) further supporting that acceleration of GlyT2 endocytosis by PKC-mediated ubiquitination directs the transporter mainly to degradation pathway.

In conclusion, our data demonstrate that in MDCK cells GlyT2 primarily resides at steady state and after constitutive endocytosis in slow recycling pathway endosomes. Ubiquitination modulates the GlyT2 turnover, in such that enhanced ubiquitination increases the transporter degradation shortening its half-life, while decreased ubiquitination promotes its stability.

### The ubiquitination status of GlyT2 in neurons is highly responsive to the free ubiquitin pool

The intracellular location of GlyT2 in recycling endosomes implies that a mechanism of deubiquitination exists that permits GlyT2 to return to the cell surface. Like many other post-translational modifications, ubiquitination is reversible and deubiquitination is accomplished by deubiquitinating enzymes (DUBs). DUBs are fundamental in the regulation of protein ubiquitination, they influence in crucial functions in the nervous system and its dysfunction is involved in some neurodegenerative diseases [26], [27], [41]. The availability of ubiquitin to target proteins towards different cellular pathways is essential to maintain the homeostasis and activity of the nervous system. Indeed, ubiquitin carboxyl-terminal hydrolases, UCHL1 and UCHL3, are abundantly expressed in the CNS, where they play important roles in the generation and modulation of free monomeric ubiquitin [26], [27], [41], [42]. Given the specific expression of GlyT2 in neurons, we used primary cultures of brainstem neurons to study the effect of inhibiting UCHL1 and UCHL3 on the ubiquitination and constitutive endocytosis of endogenous GlyT2.

**Figure 7:**
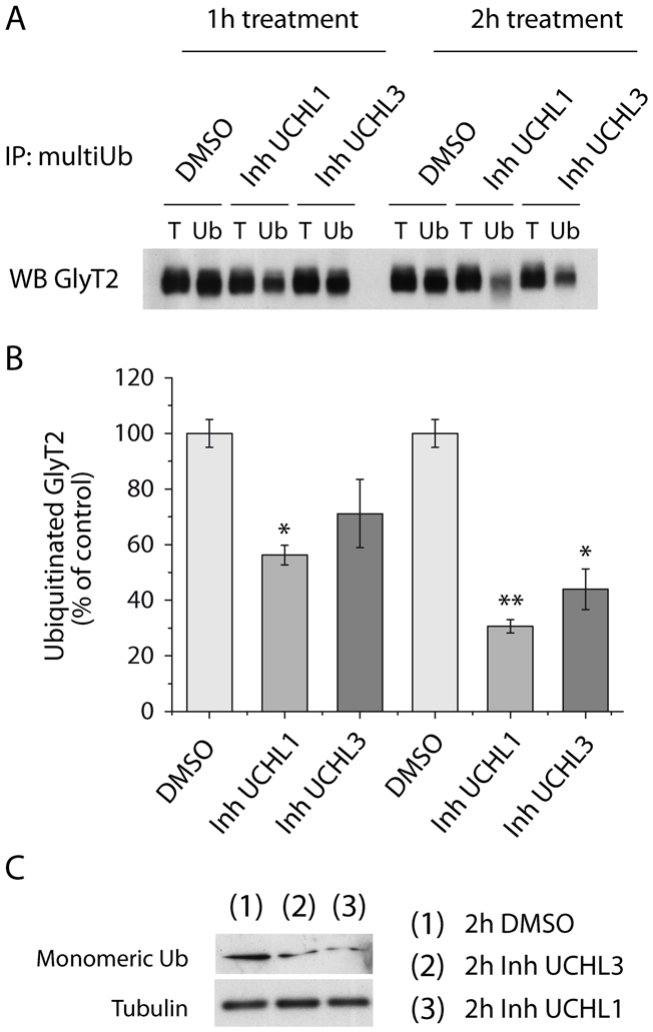
Basal ubiquitination of GlyT2 in neurons decreases when the pool of free monomeric ubiquitin is reduced. A) Brainstem and spinal cord primary neurons were treated with DMSO, UCHL1 inhibitor (10 μM) or UCHL3 inhibitor (10 μM) for the times indicated at 37°C. After lysis, the ubiquitinated proteins were immunoprecipitated with agarose-conjugated anti-multiubiquitin antibodies and the endogenous GlyT2 was analyzed in Western blots. Ub, anti-multiubiquitin immunoprecipitation (50 μg); T, total protein (5 μg). B) Quantification of three experiments performed identically to the representative experiment shown in (A). Bars represent mean ± SEM of the amount of ubiquitinated transporter relative to total GlyT2 expression. *, significant difference with respect to control, p<0.05, ** p<0.01 (ANOVA with Tukey's post-hoc test). C) Primary neurons were treated identically as in (A) for 2 h at 37°C. The cells were then harvested, lysed and whole cell lysates (40 μg/lane) were resolved by SDS-PAGE and analyzed by immunoblotting with anti-Ubiquitin antibody (clone P4D1). As a loading control, blots were reprobed with anti-tubulin.

**Figure 8:**
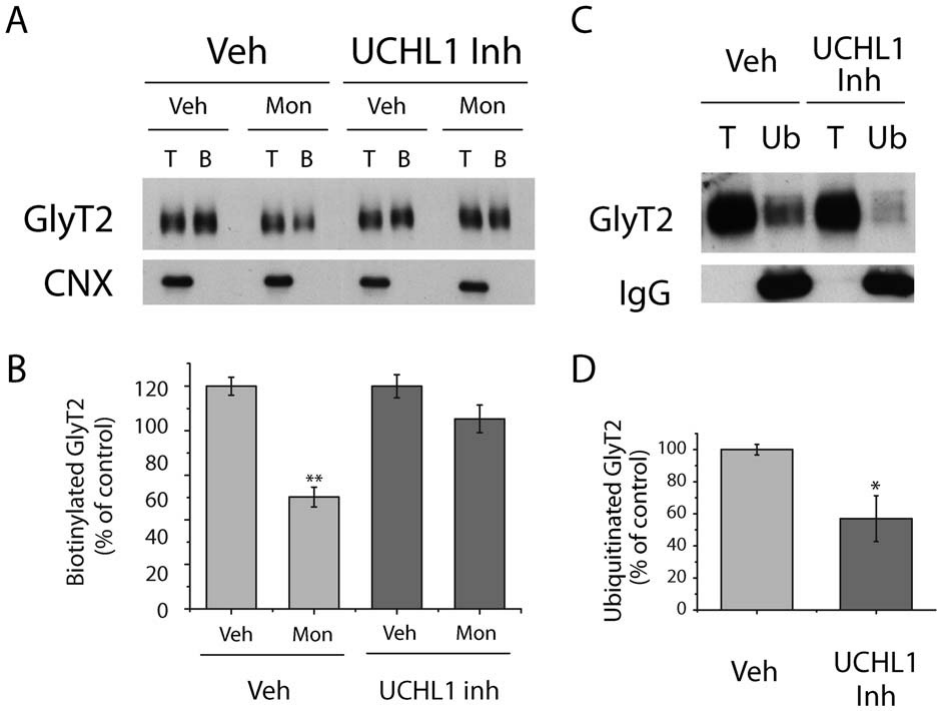
UCHL1 inhibition impairs Glyt2 constitutive endocytosis in neurons. A) Representative immunoblot of brainstem and spinal cord primary neurons. Cells were pretreated for 2 h with vehicle (DMSO) or *LDN*-57444 (UCHL1 inhibitor: 10 μM) and were then exposed to monensin (35 μM, 30 min) or the vehicle alone (EtOH), in the presence or absence of UCHL1. Cell surface proteins were labeled with sulfo-NHS-SS-biotin and the biotinylated proteins were pulled down with streptavidin-agarose beads. GlyT2 expression was analyzed in Western blots using calnexin immunodetection as a control of intracellular non-biotinylated protein. B, biotinylated protein (30 μg); T, total protein (10 μg). B) Densitometric analysis of four independent Western blots as in (A) relative to the control values (Veh). Data represent the means ± SEM. **, significant difference with respect to control; p<0.01 (ANOVA with Tukey's post-hoc test). C) Primary neurons were incubated with vehicle or *LDN*-57444 as described above, the cells were lysed and the ubiquitinated GlyT2 was immunoprecipitated with agarose-conjugated anti-multiubiquitin antibody. The immunoprecipitates were probed with anti-GlyT2 antibody. Ub, anti-multiubiquitin immunoprecipitation (75 μg); T, total protein (10 μg). D) Quantification of four experiments performed as described in (C). Bars represent the mean ± SEM level of *LDN*-57444-treated ubiquitinated transporter relative to that of ubiquitinated transporter in vehicle-treated cells. *, significant difference with respect to control; p<0.05 (Student's t-test).

Specific inhibition of UCHL1 with LDN-57444 and of UCHL3 with TCID diminished GlyT2 ubiquitination, which was more pronounced when UCHL1 was inhibited and when cells were exposed to these inhibitors for longer periods (Fig. 7A, B). The relevant control exerted by these DUBs on ubiquitin homeostasis may account for these effects since pharmacological blocking of UCHL1 or UCHL3 reduced the monomeric ubiquitin pool, in turn restricting the ubiquitination of GlyT2 in neurons (Fig. 7C). When we investigated the effect of UCH-L1 inhibition on the constitutive endocytosis of GlyT2, the decrease in biotinylated GlyT2 induced by monensin was notably attenuated after exposure to the UCHL1 inhibitor for 2 h (Fig. 8A, B), an effect that was paralleled by a significant reduction in ubiquitination of the transporter (Fig. 8C, D). Together, these data indicate a strict dependence of constitutive endocytosis on ubiquitination of GlyT2 in neurons, supporting our findings in MDCK cells. Furthermore, these results suggest that although GlyT2 seems not to be directly deubiquitinated by UCHL1 and UCHL3, both DUBs indirectly modulate the dynamics of GlyT2 ubiquitination/deubiquitination, by controlling monomeric ubiquitin homeostasis in neurons.

## Discussion

We previously demonstrated that GlyT2 is recycled between the cell surface and cell interior via constitutive and PKC-regulated clathrin-dependent endocytosis, resulting in the localization of a large proportion of the transporter in a subset of Rab11-positive endosomes in CNS nerve terminals under steady-state conditions [14], [16]. The C-terminal tail of other SLC6 family members is known to play a critical role in transporter trafficking, stability and degradation [19], [20], [22], [25], [43] and, in agreement with that, we have described an increase in the ubiquitination of GlyT2 in lysine 791 in the C-terminal tail as the major determinant of PKC-induced GlyT2 endocytosis [23]. Ubiquitination of neuronal membrane proteins controls their internalization and endocytic sorting to recycling and/or degradation pathways, and is thus an important mechanism for neuronal development and function [44]. Whereas ubiquitination is proposed as the mechanism for PKC-dependent endocytosis of several neurotransmitter transporters [17], [19], [20] its role in constitutive endocytosis appears to be specific for each transporter [19], [24], [25].

In the present study, we sought to elucidate the putative role of ubiquitination in the constitutive internalization and sorting to recycling and/or degradation pathways of neuronal GlyT2. Our results demonstrate that constitutive GlyT2 endocytosis is dependent on ubiquitination, as indicated by the decreased ubiquitination and concomitant accumulation of GlyT2 at the cell surface following inhibition of E1 ubiquitin-activating enzyme. This enzyme is the first component of the multi-step enzymatic process (E1s, E2s and E3s) that mediates the transfer of ubiquitin to lysine residues on target proteins. Indeed, substituting the C-terminal cluster of lysine residues significantly inhibited GlyT2 internalization, pointing to the K751, K773, K787 and K791 residues as major targets of ubiquitination in constitutive GlyT2 endocytosis. Similarly, ubiquitination of cytosolic lysine clusters has been implicated in the constitutive endocytosis of the glutamate neurotransporter (GLT1) [25]. The partial decrease of 4KR mutant ubiquitination suggests a basal level of ubiquitination of GlyT2, or alternatively, redundant ubiquitination of other lysines when the main conjugation sites are removed [33]. In this regard, GlyT2 sequence contains additional potential sites for ubiquitination such as six lysine residues of its long intracellular N-terminal domain, unique structural characteristic that distinguishes GlyT2 of other members of the neurotransmitter:sodium symporter family (SLC6 gene family). Further site-mutagenesis studies will be necessary to discern between these possibilities.

Moreover, the mutation of the C-terminal lysine cluster does not alter the neuronal localization of GlyT2 as indicated by the similar co-localization levels between 4KR mutant and GlyT2 wild type with the PDZ protein syntenin-1 and the SNARE protein component syntaxin1A in hippocampal neurons. Both proteins interact with GlyT2 and have been involved in the regulation of its presynaptic localization and trafficking [37], [38], [13].

In addition to promoting internalization, ubiquitination of neuronal membrane proteins can also dynamically control the post-endocytic sorting to the recycling or lysosomal degradation pathways [44]. Members of the small GTPase Rab family are known organizers of intracellular membrane protein trafficking in eukaryotic cells [39]. Our immunofluorescence studies reveal that most GlyT2 is targeted to the recycling pathway after constitutive endocytosis, reflected in the high level of co-localization of GlyT2 and Rab11, a protein that modulates slow recycling of proteins to the plasma membrane via the pericentriolar recycling compartment (or ‘long loop’: [45]). The low co-localization of GlyT2 with the late endosomal marker Rab7 after 30 min of endocytosis indicates that a small proportion of GlyT2 is targeted to the lysosomal degradation pathway. These data support our previous findings regarding the steady state localization of GlyT2 in nervous tissue [16], and demonstrate that GlyT2 undergoes continuous and efficient turnover between the plasma membrane and the intracellular compartment. The requirement of ubiquitination for constitutive GlyT2 endocytosis and the subsequent sorting demonstrated here contrasts with the pattern described for the related dopamine transporter DAT. Constitutive DAT endocytosis has been previously studied [43] and recent data have demonstrated that DAT sorting to degradation and recycling pathways occurs independently of N-terminal lysine ubiquitination [24], suggesting distinct outcomes following the ubiquitination of different membrane proteins. In addition, the analyses of the degradation rate indicated that ubiquitination negatively modulates GlyT2 turnover, as witnessed by the correlation between ubiquitination status and GlyT2 stability. Increased PKC-mediated ubiquitination therefore directs GlyT2 mainly to the degradation pathway leading to an efficient downregulation of the protein, whereas reduced C-terminal lysine ubiquitination increases its stability.

The availability of ubiquitin to target proteins in distinct cellular pathways is essential for nervous system function and DUBs contribute to ubiquitin homeostasis by recycling ubiquitin from lysosomal and proteasomal substrates. The human genome encodes nearly 95 DUBs [46], although their specific substrates and physiological roles are still poorly understood [27]. Presynaptic ubiquitin pools are particularly sensitive to variations in ubiquitin availability to be away from the site of synthesis [47]. UCHL1 is abundantly expressed in neurons and is one of the main mediators of ubiquitin homeostasis, accounting for 60% of hippocampal deubiquitination [26], [48]. The significant reduction in neuronal monomeric ubiquitin levels following pharmacological blockade of UCHL1 and UCHL3 observed in our assays and also described by other authors [26] may account for the significant decrease in GlyT2 ubiquitination described here. However, we cannot rule out the possibility that ubiquitin depletion by UCHL1 inhibition results in the up-regulation of DUBs acting on GlyT2. Compensatory mechanisms have been described in every UCH-L1 and Usp14-deficient mice where the lowered ubiquitin pool by the suppression of either DUB induced an increase in mRNA and protein levels of each other [49]. However, the rapid reduction in GlyT2 ubiquitination observed only 1 hour after of pharmacological UCHL1 blockade is not consistent with this genetic mechanism. Thus, our findings suggest that constitutive internalization of GlyT2 in neurons depends on ubiquitination, and that its ubiquitination status is highly sensitive to ubiquitin homeostasis.

It should be noted that ubiquitination is an important regulatory mechanism for inhibitory synaptic neurotransmission through control of the trafficking and turnover of glycine and GABA receptors [50]–[52]. In this regard, the modulation of GlyT2 turnover by ubiquitination described here (through endocytosis, recycling and degradation) contributes to our understanding of how inhibitory neurotransmission is regulated. As GlyT2 supplies glycine to refill synaptic vesicles in inhibitory nerve terminals, the regulation of dynamic and active GlyT2 cellular trafficking is critical for inhibitory glycinergic neurotransmission. This is evident through the disruption in transporter membrane trafficking associated with certain GlyT2 gene *(SLC6A5)* mutations associated with hyperekplexia [5], [9]. Modulation of the recruitable GlyT2 internal pool facilitates rapid and efficient neuronal adaptation to changes in synaptic neurotransmitter concentrations. Thus, GlyT2 modulation through ubiquitination increases our knowledge in the processes that control glycinergic inhibitory neurotransmission. A better understanding of the molecular mechanisms that underlie functional processes is a requirement for a more specific and precise future clinical intervention strategies in glycinergic neuromotor disorders including hyperekplexia and myoclonus, or other dysfunctions as neuropathic pain or epilepsy.
